# Structural and Biochemical Properties of Novel Self-Cleaving Ribozymes

**DOI:** 10.3390/molecules22040678

**Published:** 2017-04-24

**Authors:** Ki-Young Lee, Bong-Jin Lee

**Affiliations:** Research Institute of Pharmaceutical Sciences, College of Pharmacy, Seoul National University, Seoul 151-742, Korea; kiyoung1983@snu.ac.kr

**Keywords:** novel ribozymes, structure, catalytic mechanism, twister, twister-sister, pistol, hatchet

## Abstract

Fourteen well-defined ribozyme classes have been identified to date, among which nine are site-specific self-cleaving ribozymes. Very recently, small self-cleaving ribozymes have attracted renewed interest in their structure, biochemistry, and biological function since the discovery, during the last three years, of four novel ribozymes, termed twister, twister sister, pistol, and hatchet. In this review, we mainly address the structure, biochemistry, and catalytic mechanism of the novel ribozymes. They are characterized by distinct active site architectures and divergent, but similar, biochemical properties. The cleavage activities of the ribozymes are highly dependent upon divalent cations, pH, and base-specific mutations, which can cause changes in the nucleotide arrangement and/or electrostatic potential around the cleavage site. It is most likely that a guanine and adenine in close proximity of the cleavage site are involved in general acid-base catalysis. In addition, metal ions appear to play a structural rather than catalytic role although some of their crystal structures have shown a direct metal ion coordination to a non-bridging phosphate oxygen at the cleavage site. Collectively, the structural and biochemical data of the four newest ribozymes could contribute to advance our mechanistic understanding of how self-cleaving ribozymes accomplish their efficient site-specific RNA cleavages.

## 1. Introduction

RNA catalysts, termed ribozymes, are presumed to have existed as abundant cellular components that served a wide variety of biological functions, as well as acting as carriers of genetic information, in the “RNA world” era before the emergence of protein enzymes [1,2]. However, during the evolution of life, their numbers might have sharply declined as they competed with protein catalysts that have the chemical versatility of amino acids. Since the first discovery of RNA catalysts [3,4], much effort has been invested in the discovery and identification of new ribozymes occurring in nature and in the elucidation of their structures and catalytic mechanisms [5,6,7]. Recently, the discovery of four novel ribozymes, named twister, twister-sister, pistol, and hatchet, was made possible by the availability of an abundance of genomic sequences from all domains of life, in combination with advanced bioinformatics searching ability [8,9,10,11]. The simplest ribozyme search is based upon a systematic evaluation for architectural and sequence similarities with known ribozymes. Ribozymes have proven to carry out essential biochemical transformations, such as ribosome-mediated protein synthesis [12], RNA splicing [13], and various other RNA processing events [5,6,7]. Specifically, the large ribosomal subunit contains RNAs that catalyse the peptidyl transfer reaction, and the ribonucleoprotein complex RNase P processes the 5′ ends of tRNAs and certain other RNA substrates with the help of its catalytic RNA subunit. Some nucleolytic ribozymes are involved in rolling-circle replication of RNA genomes [3,14], co-transcriptional processing of retrotransposons [15,16,17], and the metabolite-mediated regulation of gene expressions in bacteria [18]; however, many other biological roles remain to be investigated. Of the 14 well-known ribozyme classes, nine carry out site-specific self-cleavage reactions without the help of protein chaperones or enzymes, usually through an internal phosphoester transfer using the chemistry involving general acid/base and metal ions. The nucleolytic ribozymes include hairpin [19], hammerhead [20,21], hepatitis delta virus (HDV)-like [22], glucosamine-6-phosphate synthase (*glmS*) [18], *Neurospora* Varkud satellite (VS) [23], twister [8], twister sister [9], pistol [9,10], and hatchet [9,11]. These ribozymes are characterised by divergent but similar biochemical properties, with distinctive structures adopting unique catalytic cores. The self-cleaving ribozyme classes, HDV, hammerhead, and twister, are most widely found in diverse genetic contexts and across all domains of life. The *glmS* ribozyme class members are extensively distributed in bacteria, residing in the 5′ untranslated regions of genes required for glucosamine 6-phosphate production [24,25,26]. Uniquely, the ribozyme acts as central regulator for gene expression in a negative feedback loop. It undergoes a site-specific self-cleavage only upon binding directly to the metabolite glucosamine 6-phosphate. Subsequently, this cleavage leads to the degradation of the mRNA that contains the *glmS* ribozyme, thus preventing its production [27].

During the last three years, Breaker and co-workers identified four novel classes of small self-cleaving ribozymes, termed twister, twister sister, pistol, and hatchet by using an advanced bioinformatics pipeline based on accumulating DNA sequence databases [8,9,10,11]. In particular, the ribozymes have been identified by an exhaustive genomic search for conserved RNAs near genes associated with other known self-cleaving ribozymes. The recent identification of these ribozymes has revitalized the hypothesis that many ribozymes remain to be discovered in contemporary organisms. Here, we will mainly address the structure, biochemistry, and catalytic mechanisms of these four newly identified self-cleaving ribozymes.

## 2. Structures of the Novel Ribozymes

Studies of the structures of self-cleaving ribozymes could provide a molecular platform for deepening our understanding of their biochemical properties and catalytic mechanisms. High-resolution tertiary structures, especially, could reveal the true nature of the catalytic pockets that are formed by hydrogen bonds and ionic and hydrophobic interactions. The spatial arrangement of nucleotides and metal ions in the active site is indispensable for elucidation of the chemistry involved in catalysing site-specific RNA cleavage. Inevitably, in order to remove the 2′ oxygen nucleophile that would trigger self-cleavage during crystallisation, the ribozymes were chemically modified with the substitution of deoxy (2′-H) or methoxy (2′-OCH_3_) sugars at the cleavage site. Therefore, the crystal structures trap the ribozyme in a pre-catalytic state, which is not reflective of transition states in the cleavage pathway, making it difficult to establish the reliable stepwise catalytic mechanism. In addition, it is conceivable that the substitutions distort the active site, possibly raising a doubt about the usefulness of the crystal structures.

### 2.1. Twister Ribozyme

**Figure 1:**
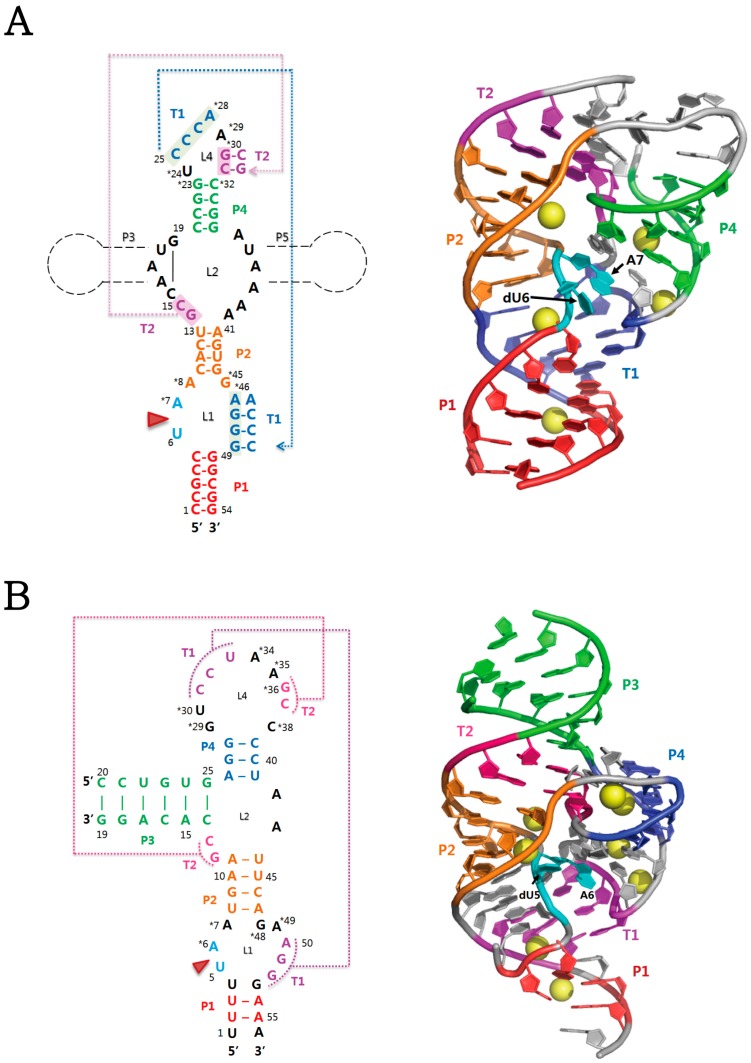
Secondary and tertiary structures of two representatives of twister ribozymes. (**A**) The structure of the twister ribozyme from *O. sativa* [30]. Additional stem-loop segments, P3 and/or P5, can be generated, as shown in black dotted lines; (**B**) The structure of the *env22* twister ribozyme [29]. In (**A**) and (**B**), red arrowhead indicates the U-A cleavage site. On the secondary structure, highly conserved nucleotides (>97%) are marked by asterisks. Stems (P1-P4) and pseudoknots (T1 and T2) are colour-coded in the tertiary structure. In particular, two nucleotides at the cleavage site and bound magnesium ions are coloured in cyan and yellow, respectively. Protein Data Bank (PDB) accession codes for (**A**) and (**B**) are 4OJI and 4RGE, respectively.

Four crystal structures of three representatives of twister ribozymes have been determined to date (PDB codes: 4OJI, 4RGE, 5DUN, and 4QJH) [28,29,30,31]. The twister structures show a similar overall fold, which is featured by the formation of two pseudoknots and co-linear stacking of helical stems. By definition, a pseudoknot is a nucleic acid secondary structure that is formed by long-range tertiary interactions through Watson-Crick base pairs between two loops [32]. There are also found obvious structural distinctions with regard to base-paring in stem P1 and organization of functional groups and a divalent metal ion in the catalytic site. The crystal structure of a basic P1 form of twister ribozyme that lacks three stems (P0, P3, and P5) of optional occurrence was solved using the Osa-1-4 sequence from *Oryza sativa* as a basis for modelling (PDB code: 4OJI) [30]. A bioinformatics search revealed that the stems, P0, P3, P5, are present in 21%, 78%, and 1% of annotated twister ribozymes, respectively [8]. The structure shows a compact fold centered about the active site containing the scissile phosphate linking dU6 and A7 (Figure 1A). This fold is formed mainly because of double-pseudoknot formation in which highly conserved nucleotides of twister mediate tertiary interactions of the terminal loop L4 with two loops L1 and L2, generating two additional helices, T1 and T2. Overall, there is a coaxial alignment of helices P1, T1, P2, and T2, which are approximately parallel to the axis of helix P4 (Figure 1A). In addition, a large helical twist between P1 and T1 is present. Specifically, the L4 nucleotides of T1 make extensive contacts with the A7 residue at the active site, which is located in the major groove of the T1-P2 helix. Other two crystal structures were determined for the *env22* twister ribozyme with 2′-H and 2′-OCH_3_ substitutions for U5 at the cleavage site, respectively (PDB codes: 4RGE and 5DUN) [29,31]. This ribozyme belongs to the P3-type twister that has the optional P3 stem. The architectures commonly show a continuous co-linear stacking of helices, such as stem P1, pseudoknot T1, stem P2, pseudoknot T2, and stem P3, from which stem P4 is projected at an angle (Figure 1B). These alignments form a compact fold centered about the U5-A6 cleavage site. These structural features are highly similar to those of another P3-type twister ribozyme, the *env9* twister (PDB code: 4QJH) [28]. The *env22* twister is different from the *O. sativa* and *env9* twisters in that it has a partially base-paired stem P1 (Figure 1B). This stem P1 is formed by U2-A55 and U3-A54 Watson–Crick pairs, whereas additional adjacent pairs such as U1-A56 and U4-G53 are not observed. Instead, U1 and U4 are involved in the formation of two base triples, U1•(U33•A50) and U4•(A34•A49), that stabilize the interaction of stem P1 with stem P2 and pseudoknot T1. In contrast, the *O. sativa* and *env9* twisters contains a fully base-paired stem P1 which prevents formation of the base triples. Notably, it remains possible that the different base-pairings of the phylogenetically conserved stem P1 affect organization of their adjoining active sites although the stem P1 is dispensable for phosphodiester cleavage [31]. 

The main differences between all the currently available crystal structures of twister ribozyme are the presence/absence of a divalent metal ion and the arrangement of key residues at the cleavage site. On the basis of these crystal structures, molecular dynamics (MD) simulations of the wild-type twisters have been carried out to derive the structure of the catalytic pocket having 2′-OH sugar of U at the cleavage site. Unfortunately, the results have shown different predictions of the transient, cleavage-competent structure and the catalytic mechanism of the twister ribozyme. In the crystal structure of the *O. sativa* twister (PDB code: 4OJI), four Mg^2+^ ions are present, but not located at the cleavage site (Figure 1A), suggesting that the metal ions participate in structural integrity rather than being directly involved in catalysis. However, the local structure surrounding the cleavage site shows that Mg^2+^ ion is coordinated to the *pro-S* non-bridging oxygen of the phosphate upstream of the scissile phosphate. This observation gives the possibility that for the wild-type twister ribozyme, this Mg^2+^ ion approaches to be positioned properly at the cleavage site, concomitant with the rearrangement of the active site nucleotides. In contrast, in the crystal structure of the *env22* ribozyme (PDB code: 4RGE), a Mg^2+^ ion is bound to the non-bridging phosphate oxygens of the U5-A6 step and its successive downstream A6-A7 step. This observation suggests that the Mg^2+^ ions neutralize and hence stabilize the developing negative charge on the scissile phosphate in the transition state of the cleavage reaction, which is consistent with the MD simulation analysis [33]. Therefore, the different binding modes of Mg^2+^ ion to the *O. sativa* and *env22* ribozymes create uncertainty in predictions of the exact roles played by divalent cations in the catalysis of twister ribozymes. In addition, the two crystal structures show a critical difference in the arrangement of the U nucleotide at the U-A cleavage site. The sugar O2′ atoms of the U of the *O. sativa* and *env22* ribozymes are positioned in a non-in-line orthogonal and near in-line alignment for an adjacent cleavable P-O bond, respectively. In general, the in-line alignment is required for productive nucleophilic attack of the O2′ oxygen to the labile phosphodiester linkage. In contrast with the U nucleotide, the conserved A nucleotide at the U-A cleavage site adopts almost identical conformations formed by extensive hydrogen bonding networks and stacking interactions. Taken together, it could be speculated that the different active site geometries are captured from the intrinsically flexible conformers of the U nucleotide with a low rotational energy barrier, and furthermore that this flexibility might facilitate the different positioning of the Mg^2+^ ions at or near the active site. Considering the crystal structure of the *env22* twister, it is likely that its active site conformation is captured under a combinatorial influence of (i) the misfolding of incompletely base-paired stem P1; (ii) Mg^2+^ ion binding; and (iii) crystal contacts.

### 2.2. Twister Sister Ribozyme

**Figure 2:**
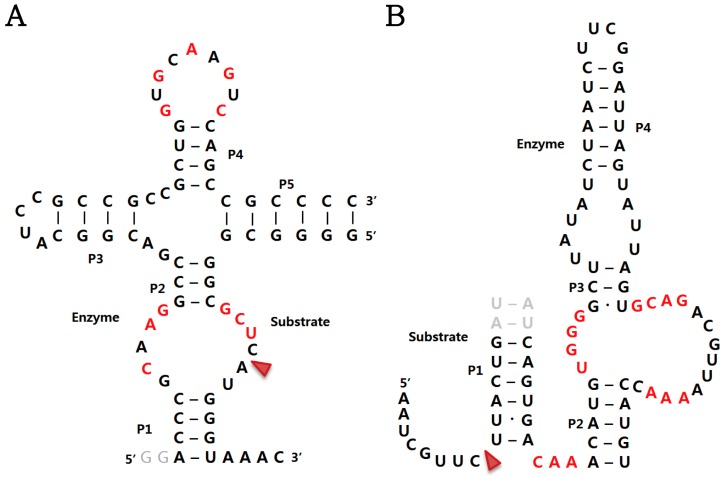
Sequence and secondary structure model of the TS-1 twister sister ribozyme [9] (**A**) and the Ht-1 hatchet ribozyme [11] (**B**). Highly conserved and non-native nucleotides are coloured in red and grey, respectively. The cleavage sites are indicated by red arrowheads.

Tertiary structures of twister sister ribozymes have not yet been determined. Consensus sequence and secondary structure model of twister ribozyme sister reveals that twister and twister sister ribozymes share some similarities in secondary structures and nucleotide sequences of their P4 terminal loops, but do not share some highly conserved key nucleotides [9]. In particular, compared to twister ribozyme, twister sister ribozyme likely has a distinct active site mainly because it cannot adopt a pseudoknot fold formed by long-range Watson-Crick base-parings of two loops (Figure 2A), which is a critical structural part that constitutes a catalytic pocket in all known twister ribozymes. In addition, there are low sequence similarities near the cleavage sites of the two ribozymes. The high-resolution tertiary structures of twister sister ribozymes will contribute to define the true structural nature of its active site, together with its catalytic strategies to accelerate RNA phosphodiester cleavage.

### 2.3. Pistol Ribozyme

**Figure 3:**
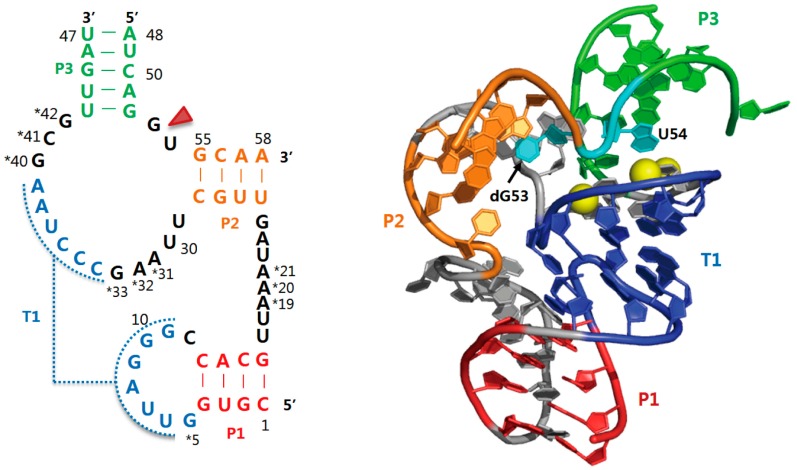
Secondary and tertiary structures of the env25 pistol ribozyme [34]. On the secondary structure (**left**); highly conserved nucleotides are marked by asterisks, and red arrowhead indicates the G53-U54 cleavage site. Stems (P1-P3) and pseudoknots (T1) are colour-coded in the tertiary structure (**right**). In particular, two nucleotides at the cleavage site and bound magnesium ions are coloured in cyan and yellow, respectively. PDB accession code is 5K7C.

Pistol ribozymes are commonly found in the Firmicutes phylum and are frequently located in the proximity of bacteriophage-related genes. There are 10 highly conserved nucleotides and many other modestly conserved nucleotides within the consensus sequence of pistol ribozymes. The conserved nucleotides likely play critical roles in nucleolytic activity, and the variable nucleotides might be associated with a slight diversity in their architectures and biochemical properties. Very recently, two crystal structures of the pistol ribozyme were determined (PDB codes: 5KTJ and 5K7C), revealing that they show similar folds with a limited secondary structure compared to other self-cleaving ribozymes (Figure 3) [34,35]. They are characterized by three stems: P1, P2, and P3, as well as a hairpin and internal loops. A six-base-pair pseudoknot helix is formed by two complementary regions located on the P1 loop and the junction connecting P2 and P3; the pseudoknot duplex is spatially situated between stems P1 and P3. Overall, the three-layered stacking of the ribozyme involves stem P1, pseudoknot stem, and a segment of stem P3 showing non-typical base-pairing, and stem P2 is positioned opposite the pseudoknot stem and contributes to an overall compact fold. The main difference between the two pistol RNAs is the kind of nucleotide 32 (guanine or adenine) at the active site. The nucleotide at this position is highly conserved as a purine [9]. However, the structures show that the G32 and A32 are located within the same position. The cleavage site of the pistol ribozyme resides in the G-U dinucleotide junction that links P2 and P3 (Figure 3), which are modestly conserved, but the length of the junction is highly conserved [34]. Notably, the G-U cleavage linkage adopted a splayed-apart conformation with in-line alignment of the 2′-OH nucleophile with respect to the to-be-cleaved P-O5′ bond. This conformation could render an unfavourable bond energy of the scissile phosphate backbone of RNA, along with the facile accessibility of intra-nucleobases and/or metal ions that participate in catalysis. In the env25 pistol ribozyme, bases A32 and G40, and hydrated Mg^2+^ ion are positioned in the proximity of the G53-U54 cleavage site [34]. However, the Mg^2+^ ion is not directly bound to non-bridging phosphate oxygens at the G53-U54 site. It is conceivable that the divalent cation contributes only to the electrostatic stabilization that promotes folding of the active site within pistol ribozyme. This could be validated by NMR spectroscopy that provides valuable information on certain base-parings in the RNA folding including pseudoknot formation, which are indicated by imino proton signals of nucleobases in a spectral width of 10–15 ppm. Based on one-dimensional ^1^H-NMR spectra, the 47-nucleotide enzyme strand of pistol is already partly pre-folded in the absence of the 11-nucleotide substrate strand and Mg^2+^ ion, and the folding can be promoted by the Mg^2+^ ion [34]. In contrast, the substrate strand exists as a linear form even in the presence of Mg^2+^ ion. The overall fold of the pistol ribozyme is completed by mixing the two strands in the presence of Mg^2+^ ion, resulting in an increased number and distribution of imino proton resonances. Another hypothesis for the Mg^2+^ ion binding in the active site is that non-cleavable modification of pistol with a lack of the attacking 2′-OH, which is required for crystallization, causes a tiny change in electrostatic potential around the cleavage site and thus inhibits a direct binding of the metal ion to a non-bridging phosphate oxygen. In the other pistol ribozyme, the G40 and a cobalt hexammine ion form hydrogen bonds with a non-bridging phosphate oxygen at the cleavage site, which may stabilize the charge of the 5′-oxygen leaving group during cleavage reaction. However, it is possible that the active site conformation is stabilized by a non-specific binding of the cobalt hexammine present in the crystallization condition. It is important to note that there is an argument about whether a divalent cation forms direct coordination with the scissile phosphate backbone of RNA. 

### 2.4. Hatchet Ribozyme

No tertiary structures have yet been determined for hatchet ribozymes, but their consensus sequence and secondary structure model can be predicted by comparing hundreds of hatchet representatives located within metagenomics DNA sequence data [9,11]. The secondary structure is characteristic of four major stems, P1-P4 (Figure 2B), and an additional P5 hairpin that is functionally dispensable. Consistent with the proposed structural model, hatchet mutants that disrupt base-parings of stems P2 or P4 show a loss of ribozyme activity, whereas compensatory mutants that restore base pairing enable ribozyme activity [11]. Highly conserved nucleotides are located in a short sequence that connects P1 and P2, and in two internal bulges between P2 and P3. These nucleotides might play critical roles in comprising the RNA cleavage site, which is consistent with the observation that mutations of two highly conserved nucleotides cause a loss of the cleavage activity [11].

## 3. Insight into Catalytic Mechanisms of the Novel Ribozymes

### 3.1. Catalytic Strategies

**Figure 4:**
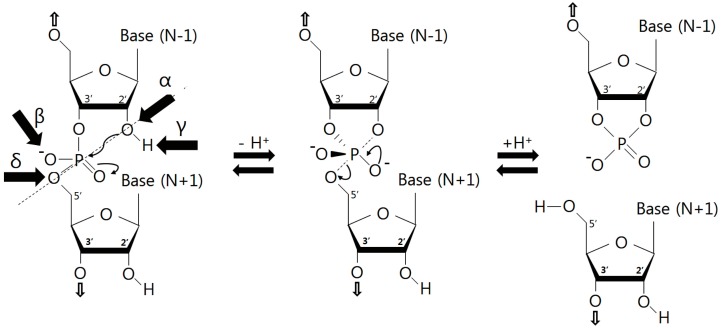
Internal phosphoester transfer mechanism for RNA cleavage. The 2′ oxygen nucleophile of a sugar attacks its adjacent phosphorus atom, together with subsequent protonation and departure of the 5′ oxygen of a sugar.

As with most other small self-cleaving ribozymes, the four recently discovered ribozymes likely follow the S_N_2-reaction mechanism for RNA phosphodiester backbone cleavage. There is general agreement that ribozymes employ an internal phosphoester transfer mechanism in which the 2′ oxygen nucleophile of a nucleotide at position +1 attacks its adjacent phosphorus atom, accompanied by the protonation and departure of the 5′ oxygen of a nucleotide at position −1 (Figure 4). Consistent with this, the substrate strands of ribozymes that lack the 2′ oxygen nucleophile are resistant to cleavage by the enzyme strand, and the reaction products include a 5′ cleavage product with a terminal 2′,3′-cyclic phosphate group and a 3′ cleavage product with a 5′ hydroxyl group. Different catalytic mechanisms would generate the different types of cleavage products. The two-metal-ion-dependent ribozymes, such as self-splicing group I and II introns, generate 5′-phosphate and 3′ hydroxyl products [36,37].

In addition, RNA cleavage mechanisms of ribozymes could be explained by either one or a combination of four molecular actions, which contribute to the catalytic rate enhancement (Figure 4) [38]: (α) the appropriate in-line orientation of the 2′-oxygen nucleophile, phosphorus electrophile, and 5′-oxygen leaving group in an active site, (β) neutralization of the negative charge of a non-bridging phosphate oxygen, (γ) deprotonation of the 2′-hydroxyl group, and (δ) neutralization and stabilization of the 5′-oxygen leaving group. The four catalytic strategies could also explain a part of internal transphosphorylation reaction of ribonuclease A among protein nucleases [39]. In particular, the in-line orientation (α catalysis) with a torsion angle of 180° is essential for an S_N_2 reaction. However, this architecture is not adopted by a canonical RNA helix. Ribozymes could provide an alternative set of stabilizing interactions to obtain this unfavorable conformation.

Ribozymes and deoxyribozymes that are created by directed evolution methods and predicted to use only two α and γ catalysis strategies, were shown to have a maximum rate constant of approximately 2 per minute under the standard reaction conditions [40]. Consistently, this rate is also near the theoretical speed limit for a nuclease on the premise that the rate enhancements for α and γ are multiplicative [38]. In particular, the cleavage rate constant could strongly rely on how much the intermediate states of the reaction are stabilized by intramolecular hydrogen-bond network or charge neutralization (e.g., by metal ions). In this regard, the rate enhancement of catalysis could be dictated by the structural arrangement of related nucleotides within the catalytic pocket, which creates a unique local electronic environment with shifted p*K*_a_ values of nucleobases in the active site. In fact, nucleolytic ribozymes adopt diverse catalytic mechanisms related to one or more functional groups of different RNA residues. 

The theoretical rate limit (approximately two per minute) for a nuclease employing the α and γ catalysis is much smaller than the rate constant (approximately 1000 per minute) of twister ribozymes [8]. Therefore, one can envision that the catalytic core of twister ribozymes can utilize either more than the α and γ strategies or a different combination of two or more of the four catalytic strategies. The crystal structures of twister ribozymes give some ambiguity for their in-line configurations (α strategy) to enhance the cleavage activity; only one of the four crystal structures shows an in-line orientation at the cleavage site. Given the fact that α strategy is indispensable for S_N_2-mediated RNA cleavage, the crystal structures at the cleavage site with non-in-line orientation may not be reflective of the true catalytic conformation. The MD simulation of the native *O. sativa* twister, including the nucleophilic 2′-OH group of U6, shows an in-line trajectory where two nucleobases, U6 and A7, sharing the scissile phosphate, are splayed-out in different directions, which contrasts with the crystal structure of the *O. sativa* twister (non-in-line orientation) [30]. The solution to this inconsistency now awaits further structural and mechanistic studies on the active site of twister ribozymes.

Twister sister ribozymes can be autonomously cleaved at nucleotides C13 and A14 on the loop connecting P1 and P2 [9]. This cleavage site is on the opposite side compared to that of twister motifs. Notably, the TS-1 twister sister shows approximately a 200-fold slower cleavage rate than twister under optimal pH and Mg^2+^ ion concentrations: their maximum apparent rate constants are approximately 5 min^−1^ and 1000 min^−1^, respectively. The pH profile and maximum rate constant for TS-1 are highly similar to those of artificial ribozymes and deoxyribozymes that might employ only two catalytic strategies (α and γ). However, two additional constructs, called TS-3 and TS-4, exhibited much higher rate constants under suboptimal pH and Mg^2+^ ion concentrations; TS-3 is predicted to have a rate constant of >100 min^−1^ under optimal reaction conditions. Taken together, the large gap between the rate constants for TS-1 and TS-3 can cause controversy about the general catalytic strategy used by twister sister ribozymes.

The pH dependence on the cleavage rate of a pistol ribozyme from *Lysinibacillus sphaericus* was assessed [10]. The pH-activity profile obtained at 25 μM MgCl_2_ showed that the rate constant increases as the pH increases: a gradient of approximately 2 between pH 5.0 and 6.25, and a gradient of approximately 1 above pH 6.25. Based on the profiles, it could be predicted that the apparent rate constant is >10 min^−1^ under physiological conditions (1 mM Mg^2+^ and pH 7.5) and >100 min^−1^ under optimal reaction conditions (Mg^2+^ concentration above 50 mM and pH 7.5–9.0) [10]. These values go over the speed limit (approximately 2 min^−1^) that has been shown for enzymes that exclusively exploit the α and γ catalytic strategies mentioned above, suggesting that pistol ribozyme employs additional β and/or δ strategies to enhance cleavage activity.

The cleavage site of the hatchet ribozyme was found to be positioned on C-U nucleotides at the base of P1 by analysing the mass peaks for the cleavage products including a 5′ product with a terminal 2′,3′-cyclic phosphate group and a 3′ product with a 5′-hydroxyl group [11]. This result suggests that hatchet exploits the general acid-base mechanism by which internal phosphoester transfer occurs, similar to many other self-cleaving ribozymes. Intriguingly, in contrast with twister and pistol ribozymes, six different natural hatchet representatives exhibit maximum cleavage rates and pH profiles similar to those of enzymes that are predicted to use only α and γ catalytic strategies, suggesting that hatchet ribozymes can exploit α and γ catalytic strategies [11]. 

### 3.2. Nucleobase Involvement in Catalysis 

Ribozymes appear to have a remarkable disadvantage in their ability to catalyze cleavage reactions when compared to protein nucleases, because RNA is deficient of functional group that has a p*K*_a_ close to neutrality, which is shown for histidine residue of protein. This functional group can be directly involved in acid- and base-catalyzed cleavage reactions at the active site. In this regard, an early assumption was that most ribozymes in nature act as obligate metalloenzymes [41]. However, this assumption has proved to be untrue. Many self-cleaving ribozymes have been proposed to use a combination of guanine and adenine nucleobases near the catalytic site as general base and acid in the cleavage reaction, respectively [6,42,43,44]. Specifically, the guanine could deprotonate the 2′-OH of the nucleotide at the cleavage site, and the adenine could neutralize the negative charge on the non-bridging phosphate oxygen at the cleavage site. The catalytic role of the guanine seems evolutionarily well conserved in self-cleaving ribozymes, compared to that of the adenine. For example, the structures of the hairpin and hammerhead ribozymes, in which a vanadate is substituted for the scissile phosphate, have revealed their catalytic transition states where the nucleotides G8 and G12, respectively, are the likely candidate for a general base to deprotonate the sugar 2′-OH of the A at the cleavage site [45,46]. Furthermore, it is notable that the G8 of the hairpin ribozyme could be involved in the transition state stabilization by forming a hydrogen bond with the non-bridging oxygen of the scissile phosphate (β catalysis). Among nine known self-cleaving ribozymes, twister, pistol, hairpin, and VS ribozymes likely exploit the guanosine amine functional groups (N2) for stabilizing the transition state during the self-cleavage [30,34,42,45,47]. As a unique feature of catalysis, the *glmS* ribozyme, which is also a riboswitch, uses the amine of a bound glucosamine-6-phosphate ligand as a general acid [25,26,48]. Moreover, the HDV ribozyme uses a cytosine nucleobase as a general acid, with the nucleophile activated by a hydrated metal ion [49,50,51]. The hammerhead ribozyme uses a metal ion-bound water as a general acid [46,52,53]. The direct participation of metal ions (usually Mg^2+^) in catalysis are also found in other ribozymes such as self-splicing group I and II introns [54,55,56,57,58], RNase P [59]. It is generally accepted that hydrated metal ions can act as a main player to position reactants, activate nucleophiles, and stabilize transition states. The catalytic strategies proposed for self-cleaving ribozymes are summarized in Table 1.

The mechanistic investigations into nucleolytic ribozymes must necessarily be supported by their extensive biochemical data. To conduct in vitro ribozyme activity assays under variable conditions, a ribozyme can be divided into two RNA strands, one of which serves as the substrate that carries the cleavage site, and the other as the enzyme that carries the catalytic RNA sequence and necessary structural elements. In particular, these constructs can be used for studying the effects of pH, divalent metal ions, and specific atomic mutations on the cleavage activity of ribozymes. The use of wild-type self-cleavage ribozyme has been limited owing to significant self-cleavage during large-scale ribozyme production that used in vitro transcription.

A P3 type of the twister ribozyme, derived from an environmental DNA sequence, was used for evaluating pH-dependent activities [8]. The results showed that rate constants increase dramatically between pH 5.0 and pH 6.3 at constant Mg^2+^ ion concentrations (50 µM), and rate constants plateaued above pH 6.5. If the two factors are independent of each other, the rate constant for this twister ribozyme in physiological conditions (pH 7.4, 1 mM MgCl_2_) would be predicted to approach approximately 1000 per minute, which is the fastest cleavage rate of those of the four recently identified ribozymes. Furthermore, significant reductions in ribozyme activity were shown for various twister mutations of highly conserved nucleotides and base-pairing nucleotides in pseudoknots and stems P1, P2, and P4. Another P3-type twister ribozyme derived from the ES ribozyme also exhibited pH-dependence on cleavage activity [30]. Notably, the pH-activity profile exhibited a bell shape with apparent p*K*_a_ values of 6.9 and 9.5. The simplest explanation for this profile is that two ionizations of the ribozyme are related to the transfer of two protons in the transition state of a concerted, general acid-base catalysis. However, reliable interpretation of such profiles can be hampered by coupling of catalytic proton transfer events with a conformational change and acid and alkaline denaturation in a variable pH [66], which can reorganize the catalytic pocket and global RNA fold and thus affect the cleavage activity of ribozyme. Structural and mutational data of the *O. sativa* twister suggest that the higher p*K*_a_ is derived primarily from residue G45, and G45 N1, which is close to the U6 O2′, acts as general base for activation/deprotonation of the nucleophile for attack at the phosphorus center during the cleavage reaction (γ catalysis) [28,30]. It is noteworthy that all four crystal structures of three representative twister ribozymes (*O. sativa*, *env22*, and *env9*) suggest a crucial catalytic role of the same conserved guanines (N1) as general base (γ catalysis), although they adopt slightly different conformations of the active site. However, the guanine N1 is expected to be fully protonated at neutral pH with a p*K*_a_ of around 10, leading to uncertainties about the catalytic role of the guanines in the proximity of the 2′-OH nucleophile. It remains possible that an energetically unfavorable enol tautomer of the guanine is transiently formed and allows the N1 to accept a proton. For the *O. sativa* and *env22* twisters, the guanine could also be involved in neutralization of the negative charge of a non-bridging phosphate oxygen at the cleavage site. The two catalytic roles of the same guanine are also found in G8 and G638 in the hairpin [67,68,69] and VS ribozymes [47,70,71], respectively (Table 1).

In addition, there is evidence suggesting that the second ionization event (p*K*_a_ ~6.5) is achieved by the N3 group of the adenine located immediately 3′ of the cleavage site [72]. The N3 is well placed to contribute to general acid catalysis (δ catalysis) by donating a proton to the 5′-oxygen leaving group [30]. This finding was somewhat unexpected as the adenosine N3 seems like a poor functional group for a general base. It is known that the major protonation site of adenine tautomers follows the order N1 (96%) > N7 (3.2%) > N3 (0.7%), which possess microscopic p*K*_a_ values of 3.63, 2.15, and 1.5, respectively [73]. However, the more basic N1 of the adenine is geometrically impossible to approach the 5′-oxygen leaving group. The spatial arrangement of the adenine in a specialized catalytic pocket might strongly influence on an increase in p*K*_a_ of its N3 group toward neutrality, which is accompanied by increased fractional protonation of the N3. Indeed, pH-dependent NMR experiments with a ^13^C2-A6 labeled, non-cleavable substrate determined the p*K*_a_ value of the adenine as 5.1, which shows a shift of 1.4 from the p*K*_a_ value of the substrate strand alone [31]. One possible origin for the raised p*K*_a_ is the exocyclic N6 of the adenine that forms hydrogen bonds with the non-bridging oxygen atoms of two successive phosphates (C25 and C26), which could stabilize the protonation state of the adenosine N3. This possibility was supported by the significantly reduced activities caused by the replacement of the adenine with guanine or 2-aminopurine, both of which do not have the exocyclic N6 [31,72]. In addition, the shift in the p*K*_a_ of active site functional groups can be caused by metal ion-mediated electrostatic modulation, as demonstrated by the pH-activity profile of the VS ribozyme showing differential modulation of nucleobase p*K*_a_ by different cations [74]. Electrostatic modulation of active residues is commonly found in many protein enzymes: for example, in serine and cysteine proteases, the p*K*_a_ of serine and cysteine residues is lowered by its neighboring positive charges [75,76].

In the case of the twister sister ribozyme, TS-1 construct, the pH-activity profile showed an increase in the nucleolytic activity over pH 5.5–7.0 and a plateau near pH 7 [9]. One explanation for this profile is that the nucleophile attack of the 2′ oxygen of a sugar is more favourable in such a condition that the 2′ position is deprotonated at a higher pH, and that the 2′ position is fully deprotonated beyond pH 7.

The crystal structures of pistol ribozymes have proposed that the A32 N3 (or G32 N3) and G40 N1 atoms are properly located to serve as a general acid and base for cleavage activity, respectively. This was well consistent with the mutagenesis studies from the env25 pistol ribozyme that single mutations of the conserved residues (A32, G40, and G42) that are adjacent to the G53-U54 cleavage site caused considerably reduced cleavage activities, compared to the wild-type control [34]. The results also support the structural data that G40·(G33-C41) base triple is an important component for the formation of a catalytic environment and that G42 positions G40 and A32 properly around the cleavage site. In particular, the A32G mutant of pistol ribozyme showed a similar cleavage activity to the wild-type, whereas the A32C mutant abolished activity, suggesting that the purine-32 N3 acts as a general acid to promote the catalysis [34]. However, considering the two pistol structures (PDB codes: 5K7C and 5KTJ), the purine N3 needs to undergo a slight conformational change to approach the 5′-oxygen leaving group at the cleavage site. In addition, ^1^H, ^13^C heteronuclear single quantum correlation (HSQC) spectra of the non-cleavable pistol with ^13^C_2_-labelled A32 obtained under varying pH conditions indicated that the p*K*_a_ value of the A32 N3 atom is approximately 4.7 [34]. The p*K*_a_ shifts by one pH unit toward neutrality, compared to the reference p*K*_a_ of adenine in the duplex and single-strand contexts. The shifted p*K*_a_ increases a chance of the protonation of the A32 N3, which could donate a proton to the 5′-oxygen leaving group at the cleavage site.

The hatchet activity increases at a constant Mg^2+^ ion concentration of 10 mM as the pH value increases up to 7.5, above which ribozyme activity plateaus. Notably, the pH-activity profile is different from the bell-shape profile of the twister ribozyme that indicates two apparent p*K*_a_ values, as described above. The simplest explanation is that the key deprotonatable group of the hatchet ribozyme is the 2′ oxygen nucleophile at the cleavage site that have a p*K*_a_ near 7, which might be shifted from its normal p*K*_a_ value of ~13.7 by unique folding of the active site. The shifted p*K*_a_ would activate the 2′-hydroxyl nucleophile, rendering it almost fully deprotonanted at neutral pH. This process is related to the γ catalytic strategy.

### 3.3. Metal Ion-Dependent Catalysis

It has been suggested that divalent metal ions serve as cofactors to promote ribozyme activities, possibly due to their abilities to compensate for the limited chemical versatility of ribonucleotide functional groups. In particular, it is possible for metal ions to neutralize the abundant negative charge of the phosphodiester backbone of ribozymes at near neutral pH, and this counterbalance could enable ribozymes to fold into their functional compact conformation [77]. The electrostatic stabilization could be explained by a combination of diffuse and specific interactions. The diffuse ion binding is achieved by a transient, non-specific metal ion coordination that forms a dynamic ionic environment with positive electrostatic potential around the RNA. In this regard, monovalent cations, which bind weakly to RNA, could also assist in proper folding and activity of ribozymes. However, it is not well established how differently monovalent and divalent cations affect the folding pathway of ribozymes. It could be assumed that cations bind differently to a variety of folding intermediates of ribozyme. On the other hand, cations could contribute to activity by long-distance electrostatic interactions with the catalytic site, especially in small ribozymes, which stabilize the negatively charged transition state and enhance electrostatic catalysis or general acid-base catalysis. Such long-range stabilization obscures the distinction between structural and catalytic roles for metal ions. In addition, the direct participation of metal ions in catalysis could include (i) assistance in sampling the correct in-line attack configuration (α catalysis) by organizing a network of nucleotides and water molecules within the catalytic pocket; (ii) contribution to rate enhancement by neutralizing the negative charge of the phosphate backbone of RNA (β catalysis); (iii) deprotonation of the 2′-OH nucleophile by a metal ion-coordinated hydroxide (γ catalysis); and (iv) protonation of the 5′-oxygen leaving group by a metal ion-coordinated water molecule (δ catalysis). Notably, it was reported that the p*K*_a_ value of a water molecule is lowered from ~16.0 in a free state to ~11.4 in a Mg^2+^ ion-bound state, which facilitates a proton transfer of the water molecule [78]. Mg^2+^ ion is one of the most common cofactors for cleavage reactions of nucleases, because of its abundance, solubility, redox stability, small size and stringent coordination geometry, compared with those of other divalent cations [79,80].

A P3 type of the twister ribozyme, derived from an environmental DNA sequence, displayed metal ion-dependent activities [8]. Cleavage rate constants for this twister construct were largely dependent on Mg^2+^ ion, displaying an increased ribozyme activity as Mg^2+^ ion concentration increased up to 1 mM, at pH 5.5. Furthermore, several other divalent metal ions also induced RNA cleavages to a similar extent as that of Mg^2+^ ion, indicating a low selectivity of metal ions. This observation could support the idea that Mg^2+^ ion plays a structural rather than catalytic role in the cleavage activity, which is consistent with the crystal structure of the *O. sativa* twister that is devoid of metal ions at the cleavage site. Real-time fluorescence experiments using fluorescent 2-aminopurine (AP) to replace U3 in stem P1 were carried out to obtain kinetic profiles for cleavage activity of the *env22* twister [29]. In principle, the fluorescence of AP would be quenched if it makes a base paring for the formation of the P1 stem. As a result, cleavage activities, reflected by the fluorescence intensity, increase as the temperature or pH value increases; for example, the activities at pH 7.5 or 20 °C are approximately 1.5-fold higher than those at pH 7.0 or 15 °C, respectively. The reaction was triggered by the addition of an excess amount of Mg^2+^ ion, suggesting that it promotes instantaneous, catalytically active folding of the ribozyme or directly participates in cleavage reaction. The latter corresponds well with the crystal structure of the *env22* twister wherein a Mg^2+^ ion is directly bound to the scissile phosphate backbone of RNA. However, there has been no clear biochemical data that support the direct participation of Mg^2+^ ion in catalytic processes when compared to the data suggesting an indirect structural role of Mg^2+^ ion in catalysis.

The binding mode of divalent metal ions to ribozymes can be probed by using cobalt hexammine, which is highly analogous to fully hydrated magnesium ion in terms of size and hydrogen bonding potential [81,82]. If a ribozyme activity is abolished in the presence of cobalt hexammine instead of otherwise essential metal ions, this could be explained by the inability of the cobalt hexamine to bind to the ribozyme in comparison with the essential metal ions that can form inner-sphere contacts in a specific binding pocket of the ribozyme. For example, cobalt hexammine can induce the nucleolytic activities of *glmS* and twister ribozymes but not twister sister and hatchet ribozymes [8,9,11,83]. The observation also suggests different metal binding sites that cause the distinct responses of the ribozymes. A key point to be considered is that the inner-sphere complexation is, in general, energetically unfavorable due to the enthalpic penalty for dehydrating the metal ion and RNA, compared to outer-sphere interactions. One strategy to overcome this penalty is the formation of the catalytic pocket in which negatively charged oxygens of nucleotides are positioned in close proximity [77]. Types of metal ions that are bound in ribozymes could have an impact on the inner-sphere complexation; Mg^2+^ ion has a relatively large propensity for outer-sphere interactions due to its small ionic radius and high charge density with a large hydration energy, compared to other divalent ions such as Ca^2+^, Mn^2+^, and Zn^2+^ [84]. This fact could suggest certain roles of Mg^2+^ ion-bound, well-ordered water molecules in ribozyme activity [85].

Another approach for examining the nature of a metal binding site is to use a comparison of reactivities of substrates that have either a phosphate group or a phosphorothioate group at the cleavage site; the latter has a sulphur atom substituted for one of the non-bridging oxygen atoms. This experimental design is based on the fact that Mg^2+^ ion commonly binds to oxygen approximately 30,000-fold more strongly than to sulphur, whereas Mn^2+^ binds to the two atoms with an equal affinity [86,87]. Therefore, if a ribozyme forms a direct, inner-sphere coordination between an Mg^2+^ ion and a non-bridging oxygen at the active site, the Mg^2+^ ion would cause a substantial loss of cleavage activity for a phosphorothioate substrate. The phosphorothioate substrates were used for cleavage assays of twister ribozyme in the presence of Mg^2+^ or Mn^2+^ ions [8]. The results showed that the two divalent metal ions have similar impacts on the cleavage activity, suggesting that metals cannot be coordinated directly to the active site of twister ribozyme. Moreover, activity assays with stereochemically specific phosphorothioate substrates were performed to separate the catalytic roles of two non-bridging oxygens, called *pro*-R and *pro*-S, at the cleavage site. The ribozyme activity is considerably decreased when the pro-R non-bridging scissile phosphate oxygen at the cleavage site is replaced with sulphur, but this reduced activity is not effectively rescued by thiophilic metal ions such as Mn^2+^ or Cd^2+^ [31,72]. These results suggest that twister ribozyme exploits β catalysis by making a contact with the *pro*-R oxygen, but this contact does not involve inner-sphere coordination of a divalent metal ion. Indeed, the structural models of the active site have proposed that the *pro*-R oxygen forms a direct hydrogen bond either with the highly conserved guanine located at the bottom of stem P2 in the *env22* and *O. sativa* twisters [30,31,42,44,72], or with the adenine of the U-A cleavage site in the *env9* twister [28]. Specifically, atomic mutagenesis revealed that the hydrogen bond between the *pro*-R oxygen and the exocyclic N2 of the guanine is the most important component to accelerate β catalysis [72]. It is therefore concluded that the reduced cleavage of the *pro*-R phosphorothioate substrate is most likely due to the impairment of the proper positioning of the exocyclic N2 with regard to the scissile phosphate. Turning the attention to the *pro-S* oxygen, the phosphorothioate substrate that replaces the *pro-S* oxygen with sulphur was used to examine the effect of the substitution on the ribozyme activity in the presence of Mg^2+^ ion [31,72]. As a result, no significant effect on the ribozyme activity was observed, implying that the *pro-S* oxygen does not serve as a ligand for Mg^2+^ ion. However, this finding is not in agreement with two crystal structures of the *env22* twister ribozyme in which a Mg^2+^ ion forms an inner-sphere interaction with the *pro-S* oxygen at the cleavage site [29,31]. To address this discrepancy, special consideration has to be given to the crystal structures that by partial base-pairings, do not fully mimic a highly conserved stem P1 adjoining to the active site, as described above. This situation probably induces a misfolded substructure near the active site, allowing a Mg^2+^ ion to bind to *pro-S* oxygen at the cleavage site.

The twister sister ribozyme can also employ divalent metal ions as cofactors for its nucleolytic activity, which is similar to many other self-cleaving ribozymes [9]. The cleavage activity strongly requires the presence of Mg^2+^ ions, which have the potential to affect the overall conformation of the ribozyme or be coordinated with its catalytic site. In particular, it shows different cleavage activities in the presence of Ni^2+^ and Sr^2+^ ions, compared to twister ribozyme; twister sister prefers Ni^2+^ rather than Sr^2+^ as a cofactor for cleavage activity, whereas twister shows the opposite preference for the two ions. Among five Group 1 monovalent cations (Li^+^, Na^+^, K^+^, Rb^+^, and Cs^+^) tested, Li^+^ ion induces the largest cleavage activity, which is similar to that of the twister ribozyme. 

Self-cleavage activities of a pistol ribozyme from *L. sphaericus* were examined at different Mg^2+^ ion concentrations [10]. The metal-activity profile obtained at pH 5.5 showed an increase in the cleavage rate with increasing amounts of Mg^2+^ ion and a near constant cleavage rate at Mg^2+^ ion concentrations above ~50 mM. It could be deduced from the profile obtained at a concentration above 1 mM that pistol ribozyme can be almost fully activated at a physiological concentration of Mg^2+^ ion (>1 mM). Eight other divalent cations were also used as cofactors for pistol cleavage assays. Of these, six cations, Mn^2+^, Ca^2+^, Co^2+^, Ni^2+^, Cd^2+^, and Ba^2+^, support the cleavage activity, suggesting that pistol activity non-specifically requires divalent cations. Two other cations, Zn^2+^ and Cu^2+^, do not promote the cleavage activity. In assays with monovalent cations, modest nucleolytic activities of pistol were shown in the presence of Na^+^ and Li^+^ ions, which have small ionic radii. Three monovalent ions, K^+^, Cs^+^, and Rb^+^, having larger ionic radii could not induce ribozyme activity. In addition, cleavage assays using phosphorothioate substitution at the non-bridging oxygen of the scissile phosphate showed that Mn^2+^ ion cannot rescued the reduced activity caused by the substitution, suggesting that a metal ion does not make inner-sphere contact with the non-bridging phosphate oxygen [10]. This is in excellent agreement with the available pistol structures that are absent of inner-sphere coordination of metal ion [34,35]. Collectively, we can conclude that pistol ribozyme do not directly involve metal ion in β catalysis. Moreover, the distribution of the divalent metal binding sites within the entire structure [35] implicates their contribution to the stable overall structural fold of pistol ribozyme, thereby increasing cleavage activity. In this situation, the N2 functional group of G40 could play an integral role in β catalysis because only this group forms a hydrogen bond with the non-bridging oxygen of the scissile phosphate.

The self-cleavage activity of hatchet was also shown to rapidly increase at pH 7.5 in the presence of increasing concentrations of Mg^2+^ ions up to approximately 10 mM, above which ribozyme activity plateaus [11]. The large dependence of Mg^2+^ ion might be due to one or a combination of structural contribution and direct participation in catalysis. Furthermore, the ribozyme activity is promoted with different levels of efficiency in the presence of some other divalent metal ions such as Mn^2+^, Co^2+^, Zn^2+^, and Cd^2+^, but is inhibited in the presence of the three metal ions, Ba^2+^, Ni^2+^, and Cu^2+^. The inhibitory effects could be explained by the displacement of Mg^2+^ ions or by distortion of cleavage-competent RNA folding. Compared to twister, twister sister, and pistol ribozymes, hatchet ribozyme appears relatively restrictive in the use of the divalent cations to enhance cleavage activity, suggesting that the hatchet ribozyme has specialized sites for metal binding by tertiary RNA folding, which could accommodate a limited number of divalent cations. The enzyme strand of the hatchet ribozyme efficiently cleaves the normal substrate in the presence of either Mg^2+^ or Mn^2+^ ions, for which rate constants are 1.5 min^−1^ and 4.6 min^−1^, respectively [11]. However, the phosphorothioate substrate is much more slowly cleaved in the same conditions, indicating that Mn^2+^ ion cannot rescue a decreased ribozyme activity for a phosphorothioate substrate. This is possibly due to a lack of direct, inner-sphere metal contact to a non-bridging phosphate oxygen at the cleavage site. 

## 4. Concluding Remarks

Over the last three years, four small self-cleaving ribozyme classes recently discovered has sparked much of the renewed interest in the structure, biochemistry, and catalytic mechanism of self-cleaving ribozymes, and related papers are already beginning to be published. Moreover, novel catalytic RNA motifs are still expected to be discovered by the ongoing development of high-throughput bioinformatics tools. The four brand new ribozymes, named twister, twister sister, pistol, and hatchet, show distinct active site architectures and divergent, but similar, biochemical properties. However, there have been some arguments about the mechanistic details underlying the self-cleavage activity of the four ribozymes although their structural and biochemical data have accumulated. This is primarily due to slight, but significant, structural differences in the active sites and to the absence of high-resolution tertiary structures. The former could be explained in terms of the spatial arrangement of key residues and the presence/absence of a divalent metal ion near the cleavage site. These differences are found in the crystal structures of the twister (PDB codes: 4OJI, 4RGE, 5DUN, and 4QJH) and pistol ribozymes (PDB codes: 5KTJ and 5K7C), respectively. The active site structures provide the information on the orientation of the 2′-oxygen nucleophile, phosphorus electrophile, and 5′-oxygen leaving group at the cleavage site. The appropriate in-line orientations responsible for α catalysis are adopted by the two pistol structures and only one of the four twister structures. Notably, the structural inconsistency of the twister and pistol ribozymes provokes thought on its plausible causes such as the influences of different P1 stem base-parings, inner-sphere metal binding, crystal contacts, and non-attacking 2′-H substitution on the active site conformation. For the twister sister and hatchet ribozymes, only the consensus sequence and secondary structure model are known [9,11]. Therefore, at present, their catalytic mechanisms could be addressed mainly on the basis of the biochemical data such as the dependence of pH, metal ions, and base-specific substitutions on ribozyme activity. These factors can induce changes in the nucleotide organization and electrostatic potential within the catalytic pocket. 

A combination of pH-dependent activity profiles, atomic mutagenesis data, and active site structures of the twister and pistol ribozymes can provide their mechanistic insights into general acid-base catalysis, possibly involving an adenine and guanine in close proximity of the cleavage site, as a general acid and base, respectively. Notably, the use of guanine as a general base is well conserved in other self-cleaving ribozymes such as hammerhead, hairpin, VS, and *glmS*, and among these ribozymes, hairpin and VS use adenine as a general acid (Table 1). In addition, there has been the subject of debate about the exact role of Mg^2+^ ion in ribozyme activity because the crystal structures of the same ribozyme have shown different coordinates of a metal ion in the active site. Two of the four twister structures show a direct binding of a divalent cation to a non-bridging phosphate oxygen at the cleavage site, as does one of the two pistol structures. Despite these structural discrepancies, comprehensive structural and biochemical data on the four novel ribozyme classes could support the idea that they use divalent cations, especially Mg^2+^ ion, to participate in structural integrity rather than being directly involved in catalysis. Among other self-cleaving ribozymes, only HDV ribozyme uses a Mg^2+^ ion as a direct contributor to β and γ catalysis [60,61]. Further structural and biochemical studies on the four novel ribozymes will be needed to address a major question about how they exploit a combination of the catalytic strategies under the assistance of nucleotide functional groups and metal ions in the specialized active site.

## Figures and Tables

**Table 1:** The catalytic strategies proposed for self-cleaving ribozymes.

Ribozyme Family	Alignment of O2′ to P-O5′ Bond (α Catalysis)	Neutralization of the Negative Charge on a Non-Bridging Oxygen (β Catalysis)	Deprotonation of 2′ Nucleophile (γ Catalysis)	Protonation of 5′ Leaving Group (δ Catalysis)	Cleavage Rate (min^−1^) at Neutral pH	Ref.
**Twister ^1^ (*O. sativa*)**	83° (4OJI)	G45	G45	A7	2.45	[30]
**Twister ^1^ (*env*9)**	83° (4QJH)	A63	G62			[28]
**Twister ^1^ (*env*22)**	148° (4RGE)	G48, Mg^2+^	G48? ^2^	A6? ^2^	2.44 (at 20 °C)	[29,31]
	91° (5DUN)			Mg^2+^-bound water? ^2^	1.41 (at 15 °C)	
**Pistol**	167° (5K7C)	G40	G40	A32 (or G32)	2.72 (at 20 °C)	[34,35]
	126° (5KTJ)				0.88 (at 15 °C)	
**HDV**	140° (5DI2)	Mg^2+^	Mg^2+^	C75	52	[60,61]
**Hammerhead**			G12	G8, metal-bound water	1.39	[46,62]
**Hairpin**		G8	G8	A38	0.1–0.5	[63,64]
***Neurospora* VS**		G638	G638	A756	1	[23,65]
***glmS***		GlcN6P	G40	GlcN6P	1–3	[26,48]

^1^ Three twister ribozymes show similar sequences and overall structures. The numbering of nucleotides is the same as those in original papers. ^2^ The question marks indicate some uncertainty about the catalytic role of the nucleobases and water molecule.
